# Supplementary material for: A Global Screen for Assembly State Changes of the Mitotic Proteome by SEC-SWATH-MS
Source: Cell Syst. 2020 Feb 26;10(2):133–155.e6. doi: 10.1016/j.cels.2020.01.001 (PMC7042714; doi:10.1016/j.cels.2020.01.001)

P26358 | DNMT1_HUMAN | DNMT1 AIM CXXC9 DNMT
Monomer MW [kDa]: 183.165 Monomer expected elution fraction: 35

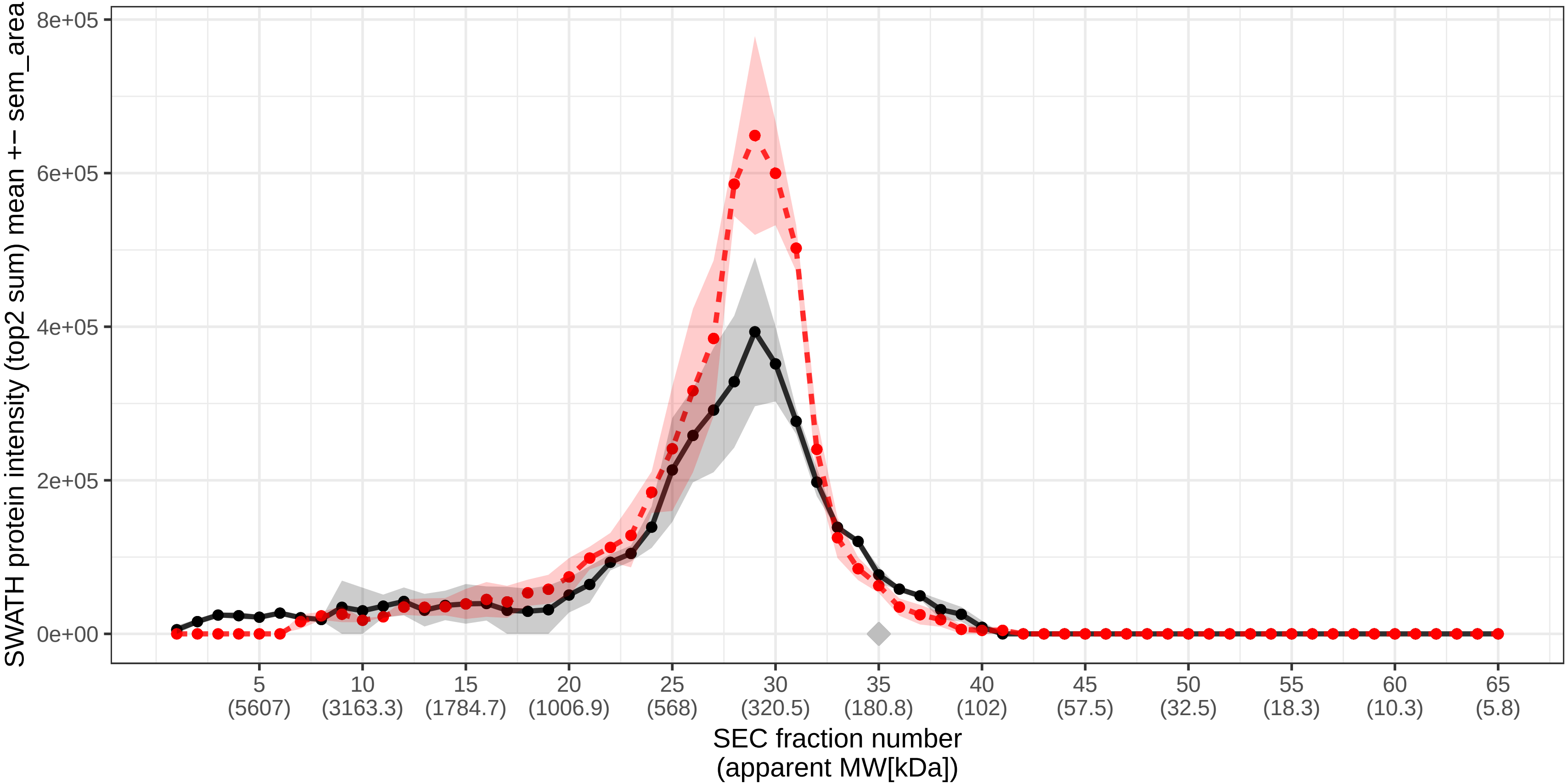

Supplement: Data S1. SEC-SWATH-MS Protein Chromatograms, Related to Figure 1 [file mmc6.zip › SECchrom_P26358_DNMT1_HUMAN_DNMT1_AIM_CXXC9_DNMT.pdf]
